# Etoposide targets 2A protease to inhibit enterovirus 71 replication

**DOI:** 10.1128/spectrum.02200-24

**Published:** 2024-11-18

**Authors:** Qinqin Liang, Sai Shi, Qingjie Zhang, Yaxin Wang, Sheng Ye, Binghong Xu

**Affiliations:** 1 Frontiers Science Center for Synthetic Biology (Ministry of Education), Haihe Laboratory of Sustainable Chemical Transformations, Tianjin Key Laboratory of Function and Application of Biological Macromolecular Structures, School of Life Sciences, Tianjin University, Tianjin, China; 2 Department of Medical and Pharmaceutical Informatics, Hebei Medical University, Shijiazhuang, China; Chinese Academy of Sciences Wuhan Institute of Virology, Wuhan, China

**Keywords:** HFMD, EV71, antiviral, Etoposide, inhibitor

## Abstract

Enterovirus 71 (EV71) is a major pathogen that causes hand, foot, and mouth disease (HFMD) in infants and children. Notably, no clinically approved drugs specifically target EV71. The EV71 2A protease (2A^pro^), a cysteine protease produced by the virus, is essential for the virus’ replication and has a significant impact on the functioning of host cells. Thus, it presents a valuable target for the discovery of antiviral medications. In this study, based on the monomers and their derivatives in the Library of Traditional Chinese Medicine (TCM), we performed virtual screening and biological experiments. We identified a derivative of a traditional herbal monomer, Etoposide, commonly isolated from the roots and rhizomes of *Podophyllum* spp. Etoposide inhibited replication of EV71 A, B, C, and CVA16 viruses in a concentration-dependent manner in a variety of cell lines with minimal cytotoxicity. Furthermore, both molecular dynamics simulations and site-directed mutagenesis assays revealed that Etoposide inhibited the activity of the EV71 2A protease by mainly binding to two residues, Y89 and P107. The findings indicate that Etoposide serves as a promising inhibitor of the EV71 2A^pro^, demonstrating strong antiviral properties and positioning itself as a formidable candidate for clinical trials against EV71.

**IMPORTANCE:**

We first used a drug screening approach focused on monomeric compounds and their derivatives from traditional Chinese medicine to identify an EV71 2A^pro^ inhibitor—Etoposide. We then performed biological experiments to validate that Etoposide suppresses the replication of the EV71 virus in a concentration-dependent manner with minimal cytotoxicity to various cell lines. Remarkably, it shows inhibitory activity against EV71 A, B, C, and CVA16, suggesting that Etoposide may be a potential broad-spectrum inhibitor. We revealed a novel mechanism that Etoposide inhibits EV71 proliferation by targeting 2A^pro^, and the interactions with Y89 and P107 are of great importance. The findings suggest that Etoposide serves as a promising inhibitor of EV71 2A^pro^, demonstrating significant antiviral properties. It stands out as a strong candidate for broad-spectrum applications in clinical research.

## INTRODUCTION

Enterovirus 71 (EV71), a novel member of the enterovirus family, is a major cause of hand, foot, and mouth disease (HFMD) in infants and children (1, 2). Approximately 70% of severe HFMD cases and up to 90% of deaths result from EV71 infection (3). Since its first isolation in 1969 in stool samples of infants with central nervous system disorders in California, USA (4, 5), EV71 has been linked to outbreaks causing widespread fatalities and injuries, resulting in severe socioeconomic impact and burden, particularly in countries and regions with EV71 endemicity in the Western Pacific (6, 7). The spectrum of diseases caused by an EV71 infection ranges from mild, asymptomatic infections in infants and children with HFMD to severe central nervous system diseases, such as aseptic meningitis, brainstem encephalitis, and neurogenic pulmonary edema (5, 8–10). The Chinese Food and Drug Administration (FDA) approved the first live, whole-virus EV71 vaccine for the treatment of severe HFMD in December 2015 (11). This is one of the few prophylactic vaccines developed against childhood infectious diseases in recent years in developing countries. However, the global application of EV71 vaccines faces challenges owing to differences in the strains prevalent in different parts of the world. Despite advances in vaccination, effective control of HFMD remains elusive, and no effective antiviral medicines exist for patients affected by the disease, including those with severe clinical phenotypes (12).

EV71 is a member of the *Picornaviridae* family (13). As a neurotropic enterovirus, it lacks a typical envelope or protrusion. Its genome encodes a polypeptide of approximately 2,194 amino acids. During EV71 replication, the encoded polypeptide is first cleaved by its own protease into three precursor proteins, designated P1–P3. Subsequently, virus-encoded 2A and 3C proteases further cleave these precursors into structural and non-structural proteins. Following translation of the polyprotein, the 2A protease (2A^pro^) initiates cleavage of the linkage sequence between VP1 and 2A (2, 14).

During the viral life cycle, 2A^pro^ is essential for the processing of viral precursor polyproteins and has a major impact on the EV71-induced apoptosis and immune evasion processes. 2A^pro^ is a cysteine protease with an active site comprising the catalytic triad H21, D39, and C110 (15). It is widely recognized that 2A^pro^ plays a pivotal role in the initial cleavage of almost all enterovirus multiprotein complexes (16). In addition, 2A^pro^ cleaves eukaryotic translation initiation factor 4G (eIF4G), interferes with host protein synthesis (5, 17), and inhibits the interferon response by cleaving the interferon receptor and the mitochondrial antiviral signaling protein (MAVS) (5, 18–20). As a major viral protein hydrolase, 2A^pro^ has emerged as a promising antiviral drug target. Owing to their low cytotoxicity and potent antiviral activity, natural plant small molecules and their derivatives are widely used as potential lead drug compounds. Identifying potent inhibitors of these natural compounds against EV71-2A^pro^ offers a promising strategy for the treatment of HFMD.

In this study, we performed drug screening on EV71 2A^pro^ using natural small molecules and identified a new inhibitor targeting EV71 2A^pro^, Etoposide, by constructing a library of Traditional Chinese Medicine (TCM) monomers and their derivatives, and subsequently applied virtual screening docking. We evaluated the antiviral potency of Etoposide by tracking the expression of EV71-GFP and VP1 in EV71-infected cells. Etoposide effectively prevented viral replication and exhibited excellent antiviral efficacy against EV71 A, B, C, and CVA16 strains across different cell lines, while demonstrating negligible cytotoxicity.

We utilized molecular dynamics simulations in conjunction with biological experiments to clarify the molecular mechanism by which Etoposide inhibits 2A^pro^. Since Etoposide was first approved by the U.S. FDA for the treatment of testicular cancer in 1983 (New Drug Application: 020906), it has been used for various cancer treatments (21), demonstrating its excellent biosafety profile. Therefore, this study expands the application of Etoposide in antiviral activity and provides new ideas for the development of anti-EV71 drugs.

## RESULTS

### Etoposide represents a novel inhibitor of EV71 2A^pro^

**Fig 1:**
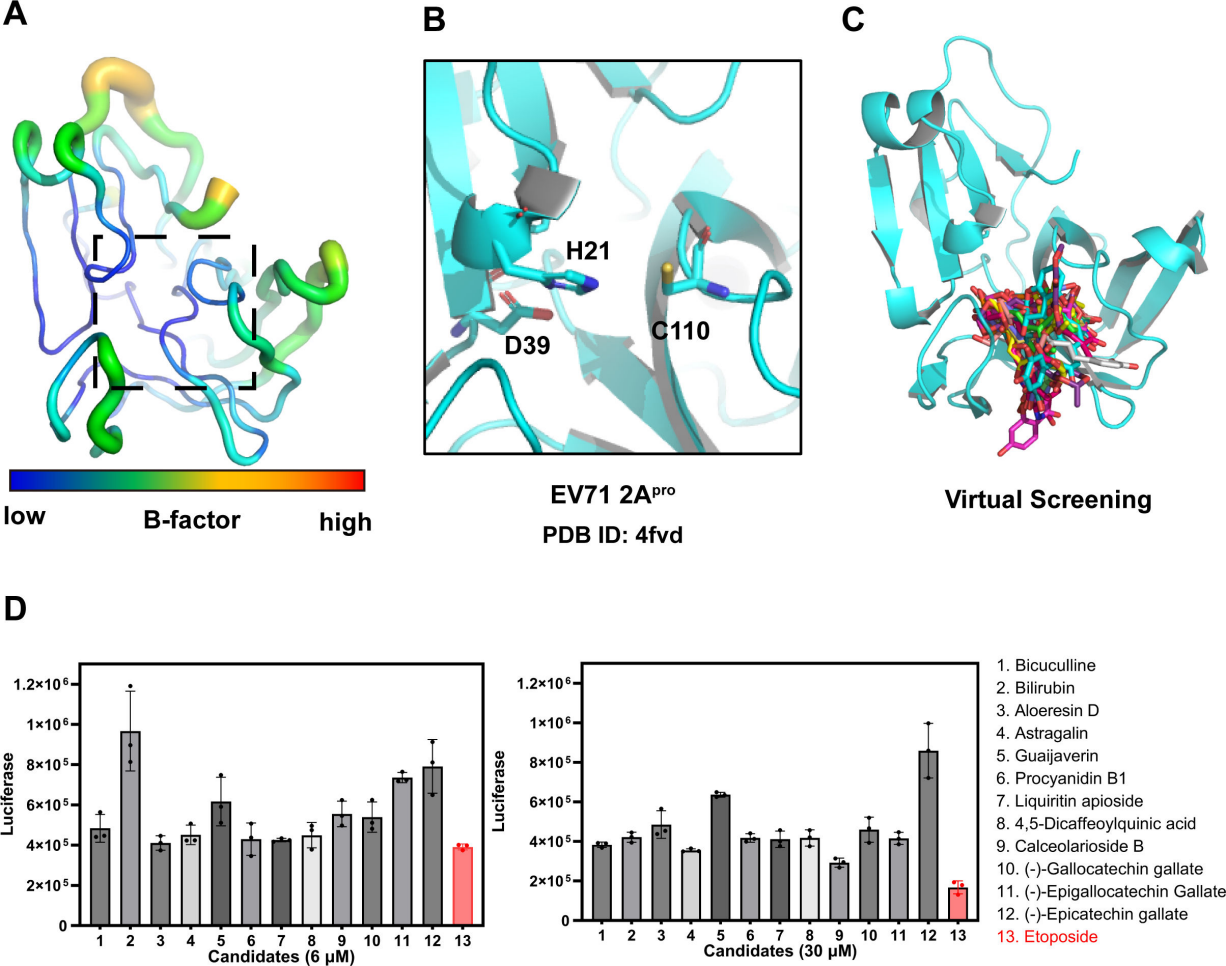
Etoposide is a novel inhibitor of EV71 2A^pro^. (**A**) EV71 2A^pro^ B-factor. (**B**) EV71 2A^pro^ inhibitor-binding pocket (PDB: 4fvd). The pocket centers (H21, D39, and C110) are shown as sticks. (**C**) Virtual screening schematic. (**D**) Inhibitory effect of 13 candidates (6 and 30 µM) on EV71 infection on RD cells. All assays were repeated three times.

To identify small antiviral molecules, we performed a virtual screening from TCM Systematic Pharmacology Database containing 2,300 monomers and derivatives (derivatives means various chemically modified versions of these monomers) (22). The receptor docking region used for virtual screening was the previously recognized 2A^pro^ inhibitor-binding pocket (PDB ID: 4fvd) (15). As depicted in Fig. 1A and B, the binding pocket exhibits strong structural stability. We performed virtual screening of the TCM monomer and their derivative library using H21, D39, and C110 as pocket centers and identified 13 candidate compounds based on binding affinity and structural diversity (Fig. 1C; Table S1).

To determine their biological activities, we further tested 13 candidates identified in the initial screening. For this, we utilized the single round pseudotype EV71 luciferase virus to characterize antiviral activity in RD cells. As the virus can replicate only once, we were able to eliminate the impact of viral reinfection. Primary screening at concentrations of 6 and 30 µM demonstrated the efficacy of Etoposide in inhibiting EV71 infection (Fig. 1D).

### Etoposide inhibits EV71 proliferation

**Fig 2:**
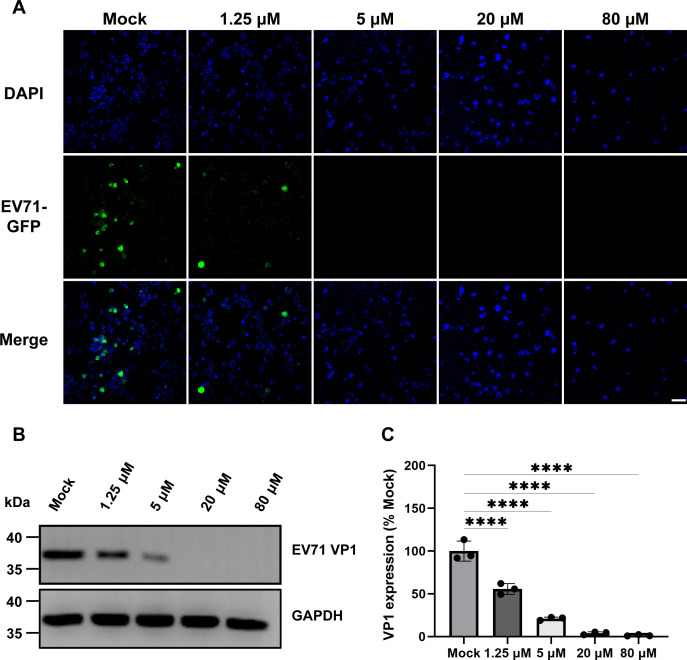
Etoposide effectively inhibited EV71 proliferation. (**A**) Concentration-dependent reduction of EV71 proliferation treated with Etoposide. RD cells were infected by EV71-GFP virus at MOI of 1, with or without treatment by various concentrations of Etoposide (1.25–80 μM) for 24 h. DAPI was used to visualize the nucleus. The GFP fluorescence signals were used to monitor viral growth. Scale bar, 75 µm. (**B**) The levels of expression of EV71 VP1 were inhibited by Etoposide in a dose-dependent reduction manner. The expression level of GAPDH was not affected by the treatment of Etoposide. (**C**) The expression of EV71 VP1 was normalized to the expression level of MOCK. Protein abundance was quantified using ImageJ software. Statistical significance of the differences between group means was evaluated by one-way analysis of variance (ANOVA) using the Tukey honestly significant difference test as a *post hoc* test (*, *P* < 0.05; **, *P* < 0.01; ***, *P* < 0.001; ****, *P* < 0.0001, N.S., not significant). All experiments were performed three times, and the representative results were shown.

To evaluate antiviral activity, phenotypic validation was performed on RD cells using the EV71-GFP virus. The phenotype validation assay demonstrated that Etoposide effectively inhibited the replication of EV71-GFP. Furthermore, the antiviral activity of Etoposide was related to the concentration, with a cytopathic effect (CPE) being observed at concentrations above 1.25 µM (Fig. 2A).

In addition, the synthesis of the EV71 viral capsid protein VP1 was characterized and found to be inhibited by Etoposide treatment, while the expression of the host housekeeping gene glyceraldehyde-3-phosphate dehydrogenase (GAPDH) was invariable. Etoposide showed an obvious inhibitory effect on VP1 expression compared with the control group (Fig. 2B and C). Together, the results support the conclusion that Etoposide effectively inhibited EV7l proliferation on RD cells.

### Etoposide affects EV71 viral replication

Time of addition assays were used to explore the infection stages that Etoposide affected. The one-round EV71 luciferase virus was employed to assess spread of virus after Etoposide treatment, thereby effectively minimizing the potential for reinfection with the virus. If the inhibitor acts at the viral replication stage, there is no significant difference in the effect between 6 h before and 8 h after dosing. NK-1.8k (23), a EV71 3C^pro^ inhibitor, serves as a control. On the contrary, if it acts during the entry phase of the virus, it only acts in the process of the virus entering the host cells, meaning that within 2 h of virus addition, it becomes less effective over time. As controls, GPP3 inhibiting viral entry were used (24).

**Fig 3:**
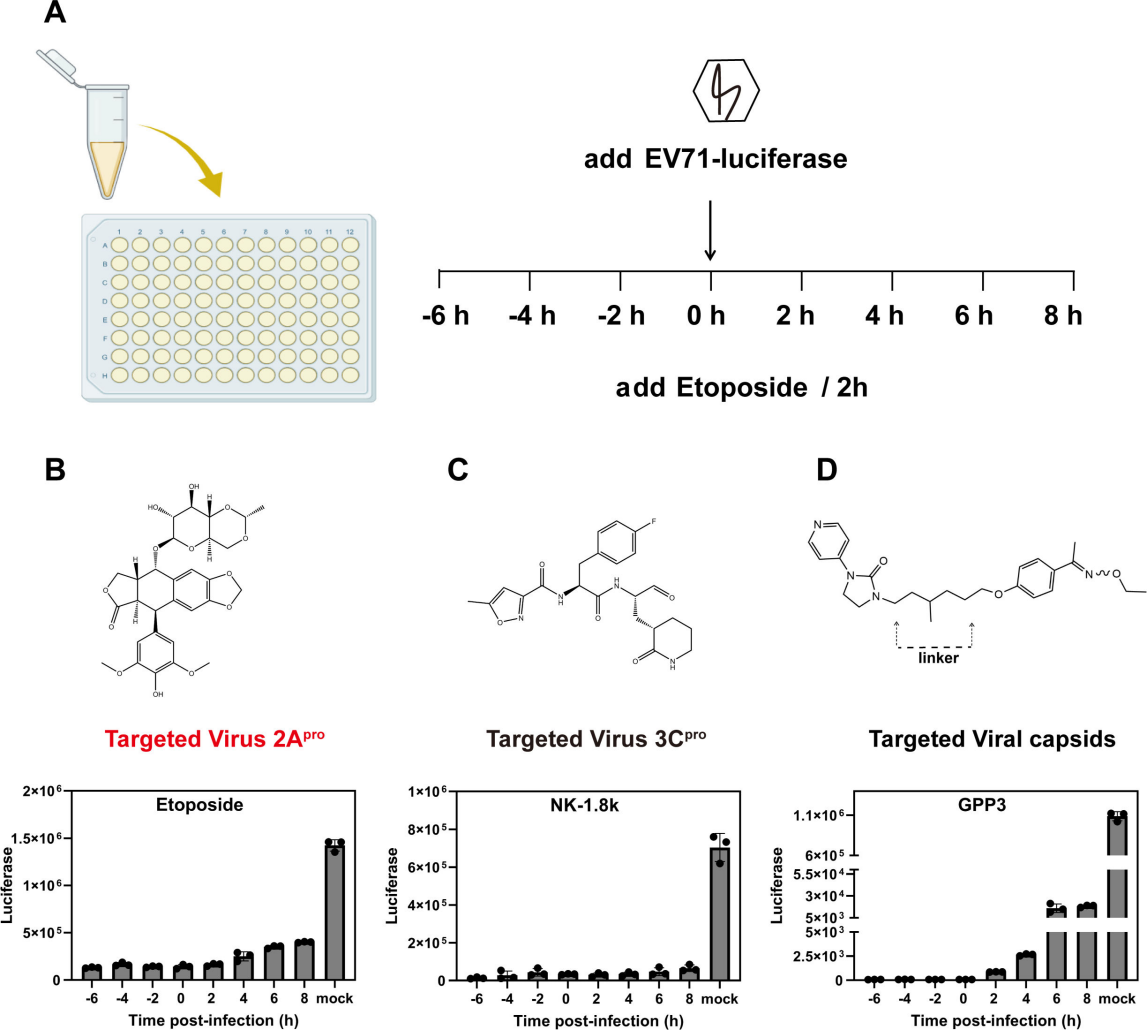
Etoposide plays inhibitory role in the viral replication stage. (**A**) Schematic diagram of time of addition assay. (**B–D**) Inhibition of EV71 luciferase reporter virus infection of RD cells by Etoposide (10 µM), NK-1.8k (2 µM) and GPP3 (0.5 µM) at various addition times (0 hpi indicates the time supplied inhibitors and virus simultaneously). Chemical structural formula of Etoposide, NK-1.8k and GPP3 are displayed above the corresponding chart. All experiments were performed three times.

As shown in Fig. 3A, EV71 luciferase reporter virus was utilized to infect RD cells, and the virus was subsequently treated with Etoposide, NK-1.8k, and GPP3 (10, 2, and 0.5 µM) at a range of time points (−6,–4, −2, 0, 2, 4, 6, and 8 h post-infection [hpi]). The time point “0 hpi” indicates the simultaneous administration of Etoposide and the virus. From −6 to 8 hpi, significant inhibitory effects of Etoposide were seen independent of the duration of treatment (Fig. 3B). This result was comparable to NK-1.8k, a viral replication inhibitor (Fig. 3C). The antiviral efficacy of GPP3 diverged from the previously mentioned result (Fig. 3D). This experiment demonstrated that Etoposide can prevent viral replication but not prevent virus entrance into host cells.

### Etoposide suppresses EV71 infection on different cell lines

**Fig 4:**
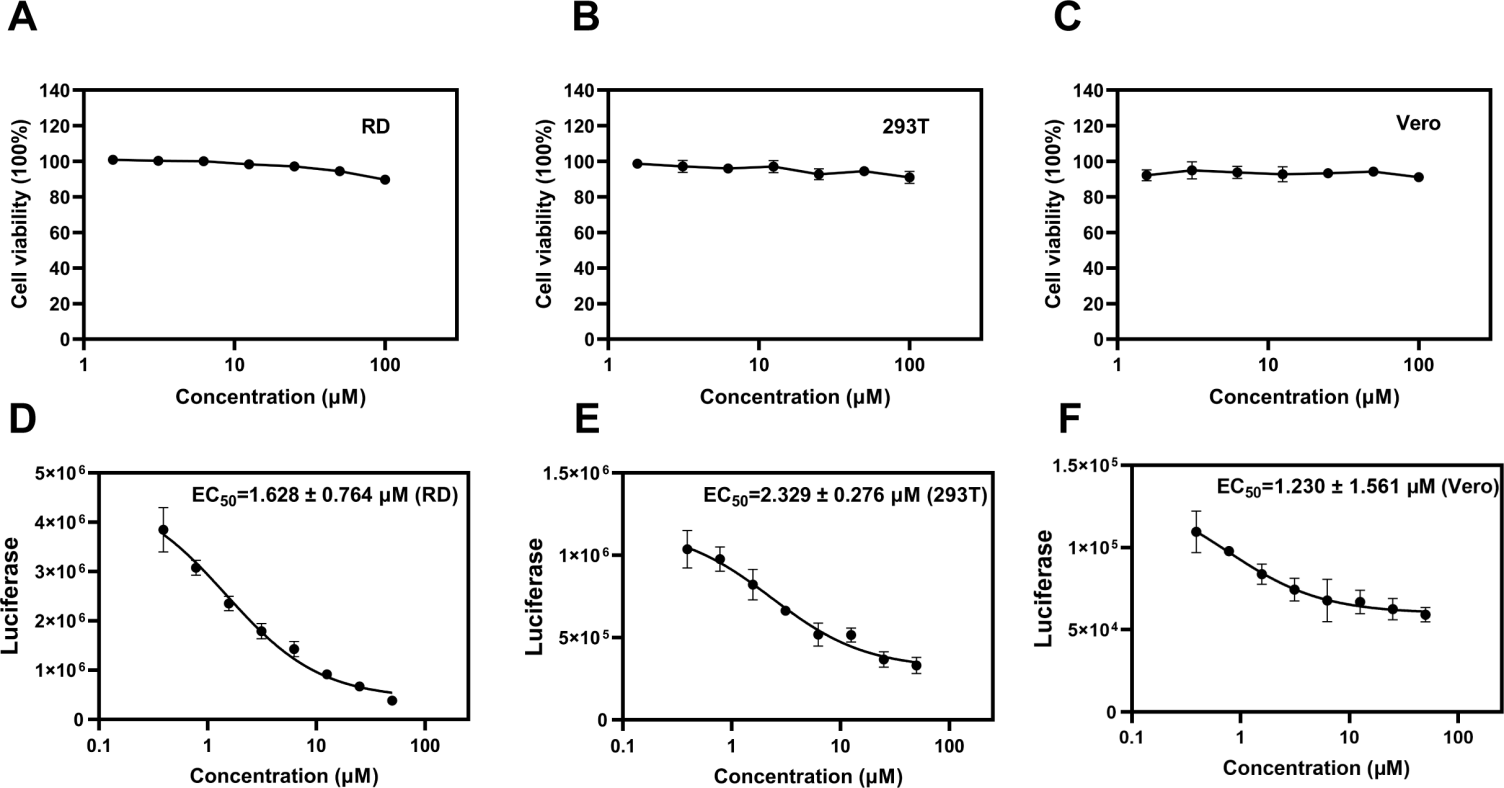
Antiviral activity of Etoposide on different cell lines. (A–C) Cytotoxicity of Etoposide on RD, HEK-293T, and Vero cell lines, respectively. (D–F) Quantification of EC_50_ on RD, HEK-293T, and Vero cell lines, respectively. All the data are means ± SD (*n* = 3).

We first examined the Etoposide’s cytotoxicity on three different cell lines. Even at a concentration of 100 µM, no apparent cytotoxic effects were seen on RD, HEK-293T, and Vero cells (Fig. 4A through C).

The EV71 virus exhibits the capacity for replication across diverse cell lines. With the aim to ascertain whether the antiviral efficacy of Etoposide is contingent upon the cell type and species, the antiviral impact of Etoposide on three cell lines was evaluated. The EC_50_ of Etoposide on RD, HEK-293T, and Vero cells was 1.628 ± 0.764, 2.329 ± 0.276, and 1.230 ± 1.561 µM, respectively (Fig. 4D through F). This suggests that Etoposide possesses an efficient inhibitory capacity against EV71 infection on various cell types. These results collectively indicate that Etoposide may serve as an effective biosafe inhibitor against EV71 infection on various cell lines.

### Etoposide is a comprehensive anti-EV inhibitors

**Fig 5:**
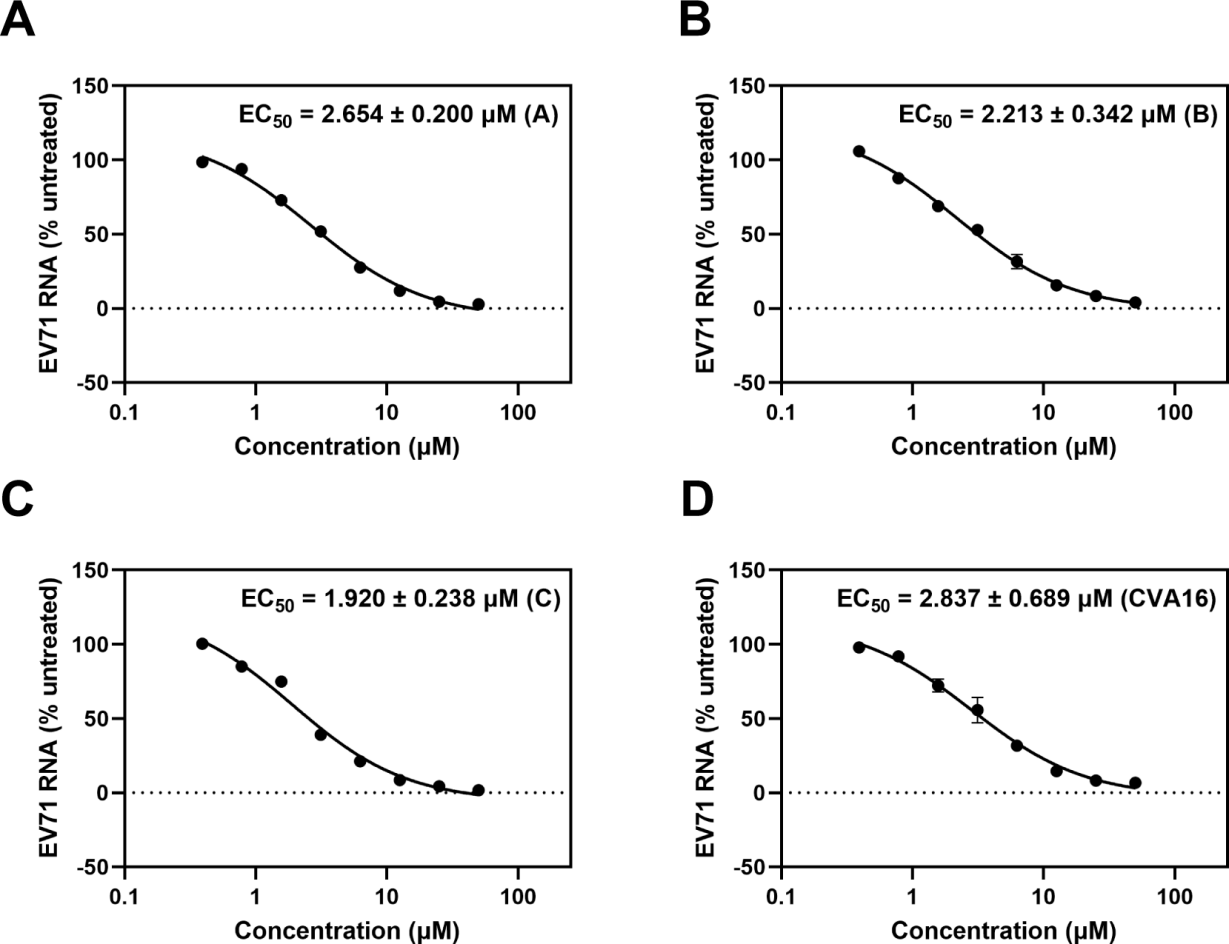
Antiviral activity of Etoposide on four viral strains. RD cells were infected with EV71 strains BrCr (A), SK-EV006 (B), and Fuyang (C), as well as CVA16 (D). The cells were subjected to a range of Etoposide concentrations, denoted as A, B, and C. The EC_50_ values were ascertained utilizing quantitative real-time PCR (qRT-PCR).

EV71 (A–C genotypes) and Coxsackievirus A16 (CVA16) are the main causes of HFMD. With the aim of ascertaining the antiviral spectrums of Etoposide, RD cells were infected with a range of viral strains, including Fuyang (genotype C), BrCr (genotype A), SK-EV006 (genotype B), and CVA16. The EC_50_ values of Etoposide on RD cells were determined to be 2.654 ± 0.200, 2.213 ± 0.342, 1.920 ± 0.238, and 2.837 ± 0.689 µM after the inhibitor treatment (Fig. 5A through D). The results showed Etoposide could be employed to treat HFMD as a broad antiviral medicine.

### Inhibition mechanism of Etoposide against EV71 2A^pro^

**Fig 6:**
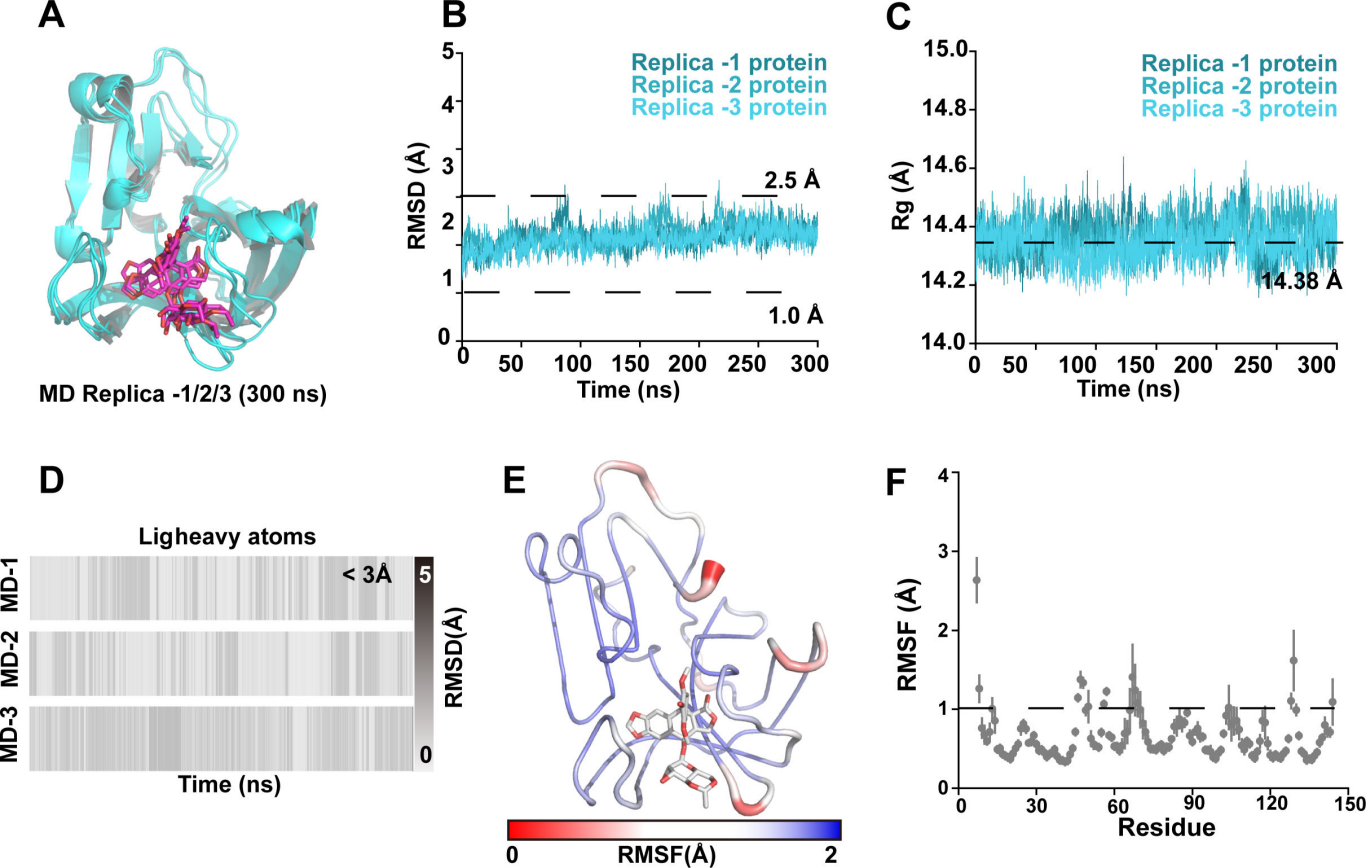
Conventional molecular dynamics simulations. (**A**) Conformational overlap of Etoposide at the 300 ns. (**B**) Root-mean-square deviation (RMSD) of protein backbone atoms. (**C**) Radius of gyration of the protein. (**D**) RMSD of Etoposide heavy atoms. (**E–F**) Root-mean-square fluctuations (RMSF) of the protein.

Conventional molecular dynamics (CMD) simulations were performed to clarify the molecular mechanism of how Etoposide inhibits 2A^pro^. The results indicated that Etoposide was stabilized in the binding pocket (Fig. 6). Etoposide’s root-mean-square deviation (RMSD) and the three trajectories’ final conformations indicated that the binding pose was consistent (Fig. 6A through C). Furthermore, Etoposide’s RMSD stabilized between 1.0 and 2.5 Å (Fig. 6D). This indicates that Etoposide can stably bind to EV71 2A^pro^. Meanwhile, RMSF of the protein showed very high stability in all regions, except the N-terminal flexibility, indicating that the protein did not produce a large conformational transition when binding to Etoposide (Fig. 6E and F).

**Fig 7:**
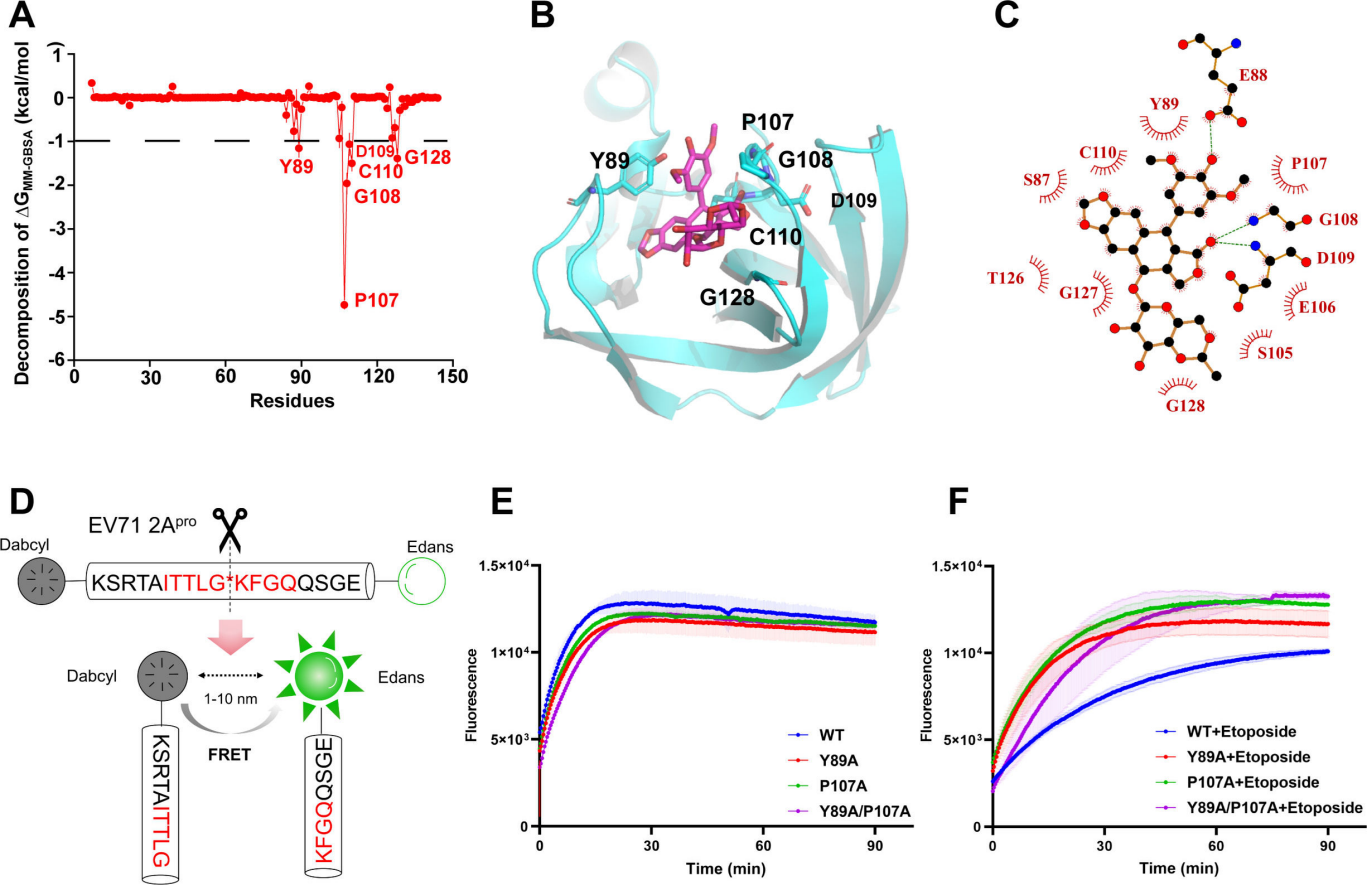
Etoposide and EV71 2A^pro^ binding mechanism. (**A**) The binding free energy decomposition of Etoposide and 2A^pro^. (**B**) The binding model of Etoposide and 2A^pro^. The 2A^pro^ is shown as cartoon, and the critical residues in the binding pocket are shown as sticks. (**C**) Interactions between Etoposide and 2A^pro^. Schematics were generated by LigPlus. The hydrophobic contacts are indicated as an “eyelash” motif. Hydrogen bonds are indicated as green dashed lines. (**D**) Schematic diagram of 2A^pro^ cleavage of a fluorescent peptide. (**E–F**) Fluorescence resonance energy transfer curves of 2A^pro^ wild-type and mutant hydrolysis substrates in the absence and presence of Etoposide, respectively. All data were performed three times.

Using the molecular mechanics generalized born surface area (MMGBSA) method, binding free energy calculations and decompositions where the residues contributed were conducted, which aimed to evaluate the quantitative effects of the affinities between 2A^pro^ and Etoposide. Etoposide bound to 2A^pro^ has a ΔG_MMGBSA_ value of −34.28 kcal/mol. As shown in Fig. 7A, labeling the residues that contributed the most favorably (less than −1.0 kcal/mol) to the binding free energy. The binding free energy decomposition spectrum indicated that Y89 (−1.15 kcal/mol) and P107 (−4.73 kcal/mol) were the two residues that considerably improved binding. Y89 and P107 are located in the “bundle” position of the binding pocket, which firmly “block off” the inhibitor so that it does not fall off (Fig. 7B). The interaction mode showed that Etoposide engaged with the 12 residues within the 2A^pro^ binding pocket interacted in various ways, including van der Waals and hydrogen bonding (Fig. 7C).

To further ascertain how Etoposide binds to 2A^pro^ and influences its enzymatic activity, FRET-based *in vitro* inhibition assays were performed utilizing the fluorescent peptide Dabcyl-KSRTAITTLGKFGQQSGE-Edans as substrates (Fig. 7D). We sequentially mutated Y89 and P107 to alanine residues. FRET analysis indicated that the mutation of Y89A, P107A, and Y89A/P107A had no significant impact on the enzyme activity of 2A^pro^ in the absence of Etoposide (Fig. 7E). However, upon addition of Etoposide, the mutant enzyme demonstrated less inhibition than the wild-type enzyme (Fig. 7F). These findings provide further evidence that Etoposide inhibits EV71 viral replication by targeting 2A^pro^ and that interactions with Y89 and P107 play a critical role.

## DISCUSSION

HFMD constitutes a grave global menace to public health. The EV71 virus stands as one of the primary culprits causing HFMD and is widely regarded as a highly hazardous neurotropic virus after poliovirus because of its high transmission capacity, wide prevalence, and neurotropism. Currently, there are no approved antiviral drugs that can act directly against EV71 infections. EV71 2A^pro^ and 3C^pro^ both play pivotal roles in EV71 viral replication, thus becoming popular drug targets. In recent years, our group has designed and synthesized a series of inhibitors to inhibit EV71 infection by targeting 3C^pro^, such as NK-1.8k (23), NK-1.9k (13), FOPMC/FIOMC (25), and SLQ-4/SLQ-5 (26). Before proceeding on to the evaluation step, these substances have to pass rigorous long-term testing and optimized alterations. In addition, a EV71 3C^pro^ inhibitor called salvianolic acid A (SA) was discovered based on structure-based drug screening (27).

Compared with 3C^pro^, 2A^pro^ remains a relatively unexploited target. Currently, several inhibitors have been identified, including the human rhinovirus 2A^pro^ peptide inhibitor (LVLQTM) (28, 29) and a synthetic derivative of the furanquinoline alkaloid CW-33 (30). These inhibitors are effective only at a concentration of 200 µM, resulting in limited use. Subsequently, notable progress was made in the field of peptide therapeutics. A peptide (GFRGKF) inhibited EV71-2A^pro^ protease activity better than CW-33 was designed based on silico strategy (31). However, peptide drugs still face significant challenges in terms of their stability, bioavailability, and delivery. As the technology of viral protease structures and their interaction with inhibitors continues to mature, the vast majority of drug studies are based on small molecules derived from natural Chinese medicines, which combine the benefits of high potency and low toxicity. These advantages can be combined to develop more effective therapies.

Here, a library of TCM monomers and their derivatives was used to screen a compound, Etoposide, for its significant inhibitory activity against EV71 infection. *In vitro* cell-based antiviral assays showed that Etoposide effectively prevented viral replication and exhibited excellent antiviral activity with EC_50_ ~2 µM across different cell lines while maintaining low cytotoxicity. In addition, Etoposide also against EV71 A, B, C and CVA16 strains. This suggests that Etoposide may be a potentially broad-spectrum antiviral drug with an effective biosafety profile in various cell lines. Molecular dynamics simulations and *in vitro* mutant fluorescence resonance experiments provide further evidence that Etoposide inhibits EV71 proliferation by targeting 2A^pro^ and that interactions with Y89 and P107 are of great importance. Identically, Y89 provides substantial free energy contributions for the peptide’s binding (31). Consistently, the structure of EV71 2A^pro^ complexed with the native substrate also showed that at the P2 position, P107/C110 and T126/G127 sandwich the L2 side chain of the substrate (32). This result suggests that the compound may block or interfere with substrate recognition and enzymatic function of 2A^pro^ by binding with Y89 and P107. Beyond its protease activity, this inhibition also plays a crucial role in lowering the viral load and stimulating an antiviral cellular response. It achieves this by blocking the cleavage of antiviral signaling proteins along with interferon receptor 1, processes facilitated by EV71 2A^pro^ (33–35). Considering the pronounced mutability characteristic of RNA viruses, we selected Etoposide-resistant strains by serially passaging EV71 through 20 rounds of culturing. Post-cultivation sequence analysis revealed a lack of mutational events within the EV71 genome, indicating that Etoposide may act as an enduring therapeutic agent (data not shown). This observation suggests that Etoposide has the potential to provide long-term efficacy against RNA viral infections, without succumbing to the common issue of resistance development.

Etoposide is a semi-synthetic derivative of epipodophyllotoxin, commonly obtained from the roots of *Podophyllum* plant species, including *P. peltatum*. It is derived from the precursor, deoxypodophyllotoxin (36). U.S. FDA first approved Etoposide in 1983, ensuring its biosafety in humans. By ensnaring the covalent Topo II-DNA cleavable complex, Etoposide inhibits DNA-topoisomerase II (Topo II) and causes DNA damage (37). It has been used to treat various types of cancer, including haematological malignancies and solid tumors (21). Additionally, non-tumoral pathologies like immune-mediated inflammatory illnesses linked to cytokine storm syndrome (CSS) are treated with etoposide (35, 38). However, there has been no evidence of its use in the treatment of EV71 viral infection until now. In conclusion, our findings indicate that Etoposide can be used to target EV71 2A^pro^ and inhibit EV71 viral replication. This provides an opportunity to facilitate research on HFMD therapy and discover novel antiviral drugs.

## MATERIALS AND METHODS

### Drug screening

The docked receptor was the protein structure of EV71 2A^pro^ (PDB ID: 4fvd) (32). Molecular docking program AutoDock Vina 1.0 was used to conduct the virtual drugs screening (39, 40). TCM database, comprising approximately 2,300 monomer and their derivatives, was screened to exclude compounds with molecular weights exceeding 700 Da or below 300 Da. Utilizing AutoDock version 1.5.6 (41, 42), we prepared PDBOT files for both the EV71 2A^pro^ and drugs. During the docking procedure, the protease was maintained as a rigid entity, whereas the ligands were allowed conformational flexibility. The grid center was strategically positioned at the epicenter of the binding pocket, defining a search volume of 20 × 20 × 20 Å^3^. A global search exhaustiveness parameter was established at a value of 50 to ensure comprehensive sampling. To capture a spectrum of potential docking conformations, we set a maximum permissible energy gap of 5 kcal/mol between the most favorable binding mode and the least favorable one.

### Cells, viruses, and compounds

Human rhabdomyosarcoma cells (RDs), human embryonic kidney 293 cells (HEK-293T), and African green monkey kidney cells (Vero) were cultured at 37°C in a humidified incubator with 5% CO_2_. The culture medium utilized was Dulbecco’s modified Eagle’s medium (DMEM), which was supplemented with 10% (v/v) fetal bovine serum (FBS), 100 U/ mL penicillin, and 100 µg/mL streptomycin. All reagents were obtained from GIBCO, USA.

The EV71 reporter virus, engineered to express either GFP or luciferase, was made as an established method (26). Professor Satoshi Koike (Tokyo Metropolitan Institute of Medical Science) generously contributed EV7l strain SK-EV006 (accession number AB469182.1), which has been genetically engineered to express green fluorescent protein (GFP) and named EV71-GFP, for initial phenotypic analysis (42, 43). A one-cycle pseudotype EV71 luciferase virus was obtained from Professor Wenhui Li (Institute of Biological Sciences, Beijing, China). This virus had the pcDNA6-FY-capsid and pEV71-Luc-replicon plasmids, which lacked the Pl region (29, 44). The EV71-GFP and EV7l luciferase viruses functioned as reporter viruses, whereas the pEV71-Luc-replicon plasmid acted as the replicon.

We obtained test molecules from Med Chem Express (MCE) and prepared it in DMSO according to their solubilities. Etoposide was dissolved in DMSO at a concentration of 50 mM, and the purity of the Etoposide was 99.94%. For Western blotting (WB), mouse monoclonal antibodies targeting EV71 VP1 protein (ab36367) were acquired from Abcam (UK), and GAPDH (HC301-01) was purchased from Trans Gen Biotech (China).

### Preliminary screening of antiviral activity

The antiviral effects of traditional Chinese medicine monomer and their derivatives (various chemically modified versions of monomers) were assessed to evaluate their inhibitory activity against EV71. In short, RD cells (3 × 10^4^) were inoculated into a 96-well plate and grown for 12 h at 37°C in a controlled environment with 5% CO_2_. Monomer derivatives (6 and 30 µM) and luciferase virus with MOI of 1 were introduced and incubated for 1 day. A microplate reader (Tecan) was used to measure the luciferase expression.

### Phenotype assay of antiviral activity

RD cells (6 × 10^5^ cells/well) were seeded in confocal dishes for 24 h. RD cells were exposed to a variety of Etoposide doses (1.25–80 μM) in the presence of EV71-GFP reporter virus. After 24 h, GFP expression was visualized and quantified using laser scanning confocal microscopy (Leica Stellaris 5, Japan).

### Western blot assay

RD cells were exposed to different concentrations of Etoposide and subsequently harvested. The cell lysates were prepared using RIPA buffer supplemented with protease inhibitors to prevent protein degradation. After the preparation of total protein lysates, they were subjected to electrophoretic separation on a 15% SDS-PAGE gel. Then, proteins were transferred to polyvinylidene fluoride (PVDF) membranes. To reduce nonspecific binding, 5% nonfat milk was used to block the membranes. Target protein-specific antibodies were used to detect the proteins of interest. Quantification of protein abundance was achieved through analysis with ImageJ software.

### Time of addition assay

The assay was performed using Etoposide, NK-1.8k and GPP3 with a view to indicating the period of inhibition of viral infection. After treating RD cells (3 × 10^4^/well) with Etoposide, NK-1.8k, and GPP3 (10, 2, and 0.5 µM), respectively, and subsequently infecting for varied lengths of time with Luciferase reporter virus. Following 24 hpi, antiviral efficacy was assessed using the Bright-Glo Luciferase substrate (Promega, USA) *via* a reduction in luciferase activity compared with the control cultures.

### Cytotoxicity of Etoposide

The cytotoxicity of Etoposide was tested in three cell lines using a Cell Counting Kit-8 (CCK-8, Beyotime). Etoposide was diluted (1.56 to 100 µM) and cultured for 24 h in a 96-well plate. Cells were incubated for 1–4 h with 10 µL of CCK-8. The absorbance at 450 nm was measured using a microplate reader (Tecan, Austria). Cell viability (%) = Etoposide-treated celluntreated cells.

### The inhibition effect of Etoposide on different cell lines

The cells were plated in 96-well plates at the following densities in per well: 3 × 10^4^ cells (RD), 2 × 10^4^ cells (HEK-293T), and 3 × 10^4^ cells (Vero). The plates were then cultured overnight at 37°C with 5% CO₂. The cell lines were treated with Etoposide at concentrations varying between 0.05 and 50 µM. After 2 h, luciferase reporter virus with MOI = 1 was introduced, and it was incubated for 1 day. Following incubation, the supernatant liquid was carefully aspirated, and cell lysis was induced using Bright-Glo Luciferase substrate. A microplate reader was used to measure the luciferase activity. GraphPad Prism 9 was used to determine the EC_50_.

### Antiviral activity on different viral strains

To investigate the potential of Etoposide as a broad-spectrum inhibitor, EC_50_ assays were performed on several viral strains. As mentioned previously, RD cells (1.5 × 10^5^ per well) were given different doses of compound (0.05–50 µM) for 2 h prior to infection with EV71 (A, B, C) and CVA16. The RNA of virus was subsequently measured using qRT-PCR to ascertain the effective concentration of EC_50_.

### Quantitative, real-time PCR (qRT-PCR)

Viral genome replication suppression following chemical treatment was measured using the EV71 C virus (Fuyang strain). RD cells were plated at 1.5 × 10^5^ cells/well and incubated at 37°C for 1 day. The cells were then treated with serially diluted Etoposide and the virus. At 24 hpi, RNA was extracted using TRIzol (TransGen, China) for high-efficiency RNA yield. The RNA was subjected to qRT-PCR using a SYBR Green RT-PCR kit from Bio-Rad (USA) targeting the 5′ UTR of EV71 and the housekeeping gene GAPDH. ΔΔCT method was used to quantify the transcript levels to assess the relative gene expression.

### Molecular dynamics simulations

The tleap program Amber 22 was used to build the simulation systems (45). About approximately 25,000 atoms were contained in the simulation boxes. The specific protocol was according to the previous method (27). To calculate the statistical distributions, snapshots were taken from all equilibrium molecular dynamics (MD) trajectories every 100 ps. The consequent trajectories were analyzed using the Amber 22 software’s CPPTRAJ module. For additional analysis, the AmberTools22 package’s MMPBSA.py (46, 47) was used to evaluate the free energies of the two complexes. Subsequently, 100 conformations (between 200 and 300 ns) from each equilibrious trajectory were used for the computations.

### Protein expression and purification

The EV71 strain (GenBank accession no. EF373576) was used as the basis for the original synthesis of the cDNA encoding EV71 2A^pro^. The cDNA fragment was then inserted into the pGEX-4T-2 vector (GE Healthcare), thereby generating a plasmid that encodes a fusion protein comprising the N-terminal glutathione S-transferase (GST) and 2A^pro^ sequence. The vector pGEX-4T-2–2A^pro^ was used to transform the *Escherichia coli* strain BL21 (DE3) pLysS (Novagen). After the bacterial cultures reached OD_600_ = 0.6 at 37°C, 1 mM isopropyl-β-D-thiogalactoside (IPTG) was added to the cultures and left to promote 2A^pro^ expression for 12 h at 16°C. The lysis buffer, which contained 20 mM Tris-HCl and 100 mM NaCl, pH 7.5, was used to collect and ultrasonicate bacterial cells. Cell debris was removed from the lysate by centrifuging it for 45 min at 15,000 rpm. A glutathione–sepharose column was loaded with the supernatant. Following multiple column volumes of buffer washing, the GST tag was removed overnight at 22°C using thrombin in lysis buffer. A Superdex 75 16/60 column (GE Healthcare) with lysis buffer was used to further purify the elution, and then the peak of 2A^pro^ was collected for further analysis.

### *In vitro* inhibition assay

The fluorescent peptide dabcyl-KSRTAITTLGKFGQQSGE-Edans served as the substrate. The assay mixture contains EV71 2A^pro^, substrate, and Etoposide (5, 20, and 20 µM) in lysis buffer at 30°C. Using a microplate reader, the fluorescence intensities were measured every minute for 90 min at λ_ex_ = 340 nm and λ_em_ = 500 nm.

### Data analysis

Data analysis and graphical display were carried out with GraphPad Prism 9. The data are given as the mean ± standard deviation (SD). Statistical significance was established at *P* ≤ 0.05. (*, *P* < 0.05; **, *P* < 0.01; ***, *P* < 0.001; ****, *P* < 0.0001). Non-covalent interactions between the binding pocket and ligand were examined using LigPlus.

## Data Availability

The data supporting the findings of this study are available from the corresponding authors upon reasonable request.

## SUPPLEMENTAL MATERIAL

Table S1 spectrum.02200-24-s0001.docx Affinity assessment of candidates.

ASM does not own the copyrights to Supplemental Material that may be linked to, or accessed through, an article. The authors have granted ASM a non-exclusive, world-wide license to publish the Supplemental Material files. Please contact the corresponding author directly for reuse.
