# Supplementary material for: Etoposide targets 2A protease to inhibit enterovirus 71 replication
Source: Microbiol Spectr. 2024 Nov 18;13(1):e02200-24. doi: 10.1128/spectrum.02200-24 (PMC11705958; doi:10.1128/spectrum.02200-24)
Supplement: Table S1 — Affinity assessment of candidates. [file spectrum.02200-24-s0001.docx]

**Supplementary information of “Etoposide targets 2A protease to inhibit enterovirus 71 replication”**

Qinqin Liang^a,#^, Sai Shi^b,#^, Qingjie Zhang^a^, Yaxin Wang^a,^*, Sheng Ye^a,^*, Binghong Xu^a,^*

*^a^ Frontiers Science Center for Synthetic Biology (Ministry of Education), Haihe Laboratory of Sustainable Chemical Transformations, Tianjin Key Laboratory of Function and Application of Biological Macromolecular Structures, School of Life Sciences, Tianjin University, Tianjin, P.R. China;*

*^b^ Department of Medical and Pharmaceutical Informatics, Hebei Medical University, Shijiazhuang 050017, China*

*Corresponding authors:

Yaxin Wang, School of Life sciences, Tianjin University, Tianjin 300072, China, Email: [wangyaxin@tju.edu.cn](mailto:wangyaxin@tju.edu.cn);

Sheng Ye, School of Life sciences, Tianjin University, Tianjin 300072, China, Email: [sye@tju.edu.cn](mailto:sye@tju.edu.cn);

Binghong Xu, School of Life sciences, Tianjin University, Tianjin 300072, China, Email: binghong_xu@tju.edu.cn;

^#^ These authors contribute equally to this work

**Supplemental Table 1**

**Table 1. Affinity assessment of candidates**

| Candidates | Binding energy (kcal/mol) |
| --- | --- |
| Bicuculline | -10 |
| Procyanidin B1 | -9.2 |
| Liquiritin apioside | -9.2 |
| Aloeresin D | -9.1 |
| Astragalin | -9.2 |
| 4,5-Dicaffeoylquinic acid | -9 |
| Guaijaverin | -9.7 |
| Calceolarioside B | -9.5 |
| (-)-Gallocatechin gallate | -9.5 |
| (-)-Epigallocatechin Gallate | -9.3 |
| Bilirubin | -9.4 |
| (-)-Epicatechin gallate | -9.6 |
| Etoposide | -9.9 |
